# Supplementary material for: Perceptions of adolescent mothers towards adolescent repeat childbirth in Soroti district, Teso sub-region, Uganda: A phenomenological study
Source: PLoS One. 2023 Apr 27;18(4):e0268612. doi: 10.1371/journal.pone.0268612 (PMC10138832; doi:10.1371/journal.pone.0268612)
Supplement: Attachment — Submitted filename: Response to Reviewers..docx [file pone.0268612.s003.docx]

[mulaposhi@gmail.com](mailto:mulaposhi@gmail.com)

Dated: 6/2/2023

Dear Reviewers,

RE: **Responses to Reviewer’s comments**

Greetings!

I thank you so much for sparing your valuable time to review this article. I would wish to let to your attention that these comments have been very impactful to this manuscript and have been taken positively.

Kindly receive them in this tabulated form below;

| **Statement in the manuscript** | **Comment** | **Response** | **Reference** |
| --- | --- | --- | --- |
| We also recommend further research to be done in Uganda, especially in other districts of Teso region to validate our findings. | How? Will it be a quantitative or still qualitative? This is not completely clear. | I strongly agree with the reviewer. The recommendation does not apply to this study since its a qualitative study. Therefore, this recommendation has been deleted. | Abstract section, and Conclusions and Recommendation section |
|  | The justification for the design used is imperative and it was not given. | This was a phenomenological study to capture the lived experiences of mothering a repeat childbirth in adolescence. Focus group discussions (FGDs) were conducted to collect textual data regarding the perceptions of adolescent mothers aged 15 to 19 years with repeat childbirth towards adolescent repeat childbearing. | Study design section |
|  | Information below does not the tell the type of sampling that was utilized. | Upon identification of the potential participant, the research team would mobilize her. The identified participant was explained to the study details, asked for consent on voluntary basis.  Once she consented, she would be issued an invitation letter, indicating the date, time and venue where the interview was to take place. She would then refer the researcher to another adolescent mother mothering a repeat childbirth (snowball sampling used, because of difficulty of identifying such participants due to the stigma associated with adolescent repeat childbirth). This was done until when all the required 8 participants were enrolled for an FGD. Prior to commencement of the FGD, consent was again sought from the participants for their participation, and to record their voices. The process was repeated to achieve the required number of participants for the next FGD. This was continued until when theoretical saturation was achieved (a point in time when no new information was generated).  Theoretical saturation of the data was achieved with 3 FGDs (24 participants).  **Snowball sampling was used.** | Procedure section |
| Whether the research ethics committee or IRB specifically waived the need for their consent. |  | The participants being emancipated minors, the IRB/ Research ethics committee waived off the need for their consent. |  |
|  | How was the interview guide developed? | The interview guide was developed in collaboration with a team of experts on Adolescent Health of Busitema University, Faculty of Public Health. The study tool was piloted and re-adjusted with the guidance of Supervisors at Busitema University Faculty of Health sciences, and Higher Degrees committee of Busitema University before being rolled out. | Data collection tool section |
|  | When was the study conducted? | October, 2020 | Study area/study setting |
|  | Was the pilot interview done? | Yes | Data collection tool section |
|  | What was the outcome from the pilot interview? | The outcome from the pilot study informed the interview guide and some of the results are similar to the study findings presented in the result section. | Data collection tool section |
|  | How was nonverbal communication captured? | The data collection team took notice of the participants’ mood, friendliness, body language and intonations. | Data collection section |
|  | Was the focus group interview recorded? | Yes | Data Management |
|  | What question were posed to the participants? | The questions posed focused on modified socio-ecological model regarding the factors associated with repeat childbirth. These were regarding adolescent mothers’ perceived individual factors of repeat childbirth, factors related to the sexual partner of the adolescent mother, adolescent mother’s family related factors and factors related to the peers and community of the adolescent mothers. |  |
|  | Since this study was a qualitative research based on a phenomenology, i expect to see four principles of trustworthiness as proposed by Guba and Lincoln namely: Credibility, dependability, transferability and confirm-ability. | To attain confirmability, the findings ensured precision and accuracy by involving other researchers, Faculty board, and higher degrees Committee of Busitema University. The researcher had a bias on data relating with repeat childbirth. Other Researchers were asked to read and react to transcripts, with their embedded researcher Interpretation so as to achieve dependability. To attain credibility, data triangulation using data from transcripts and audio recordings was done. Also different members of the research team analyzed the findings. | Data collection methods section |
|  | Ethical approval numbers should be clearly indicated here. | MRRH-2020-18 | Ethical consideration section |
|  | Can the author have a clear section on (1) Characteristics of study participants,  (2) Summary of themes and sub themes that emerged from data analysis  And  Clear presentation of findings with clearly stated themes and sub themes.  As of now themes are not clear for reader to follow.  They can be bold or numbered. | This has been done | Results section |
|  | This is not completely clear. | I strongly agree with the reviewer. The recommendation does not apply to this study since its a qualitative study. Therefore, this recommendation has been deleted. |  |

Looking forward to hear from you,

Sincerely yours

Posiano Mulalu
